# Supplementary material for: Stem Cell-Induced Inflammation in Cholesteatoma Is Inhibited by the TLR4 Antagonist LPS-RS
Source: Cells. 2020 Jan 14;9(1):199. doi: 10.3390/cells9010199 (PMC7017370; doi:10.3390/cells9010199)
Supplement: Supplementary file 1 [file cells-09-00199-s001.pdf]

cells

MDPI

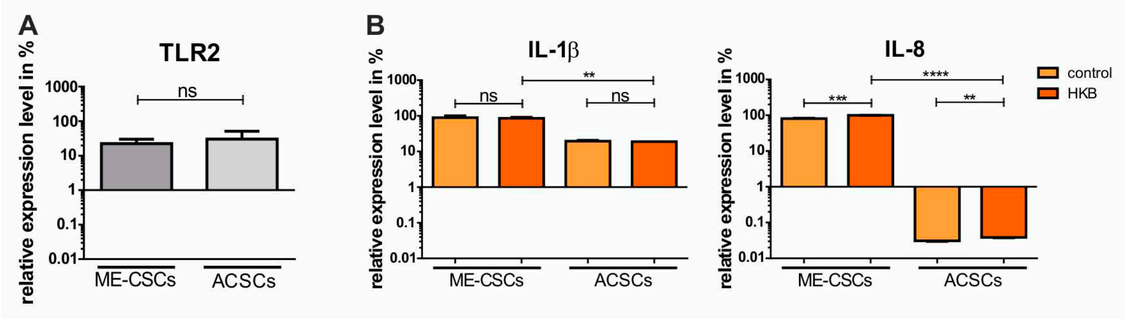

Figure S1. Inflammatory signaling in ME-CSCs is not mediated via TLR2. (A) qPCR analysis revealed no significant changes in the expression of TLR2 between ME-CSCs and ACSCs (mean of the relative expression levels from ME-CSCs and ACSCs , n=3, ns>0.05, Mann Whitney test, one-tailed, confidence interval: 95%) (B) Expression levels of IL-1ß and IL-8 in ME-CSCs and ACSCs were not affected by exposure to heat killed *S. aureus* (HBK) (mean of the relative expression levels from ME-CSCs and ACSCs, n=3, ****≤0.0001, ***≤0.001, **≤0.01, ns>0.05, unpaired t-test, one-tailed, confidence interval: 95%).

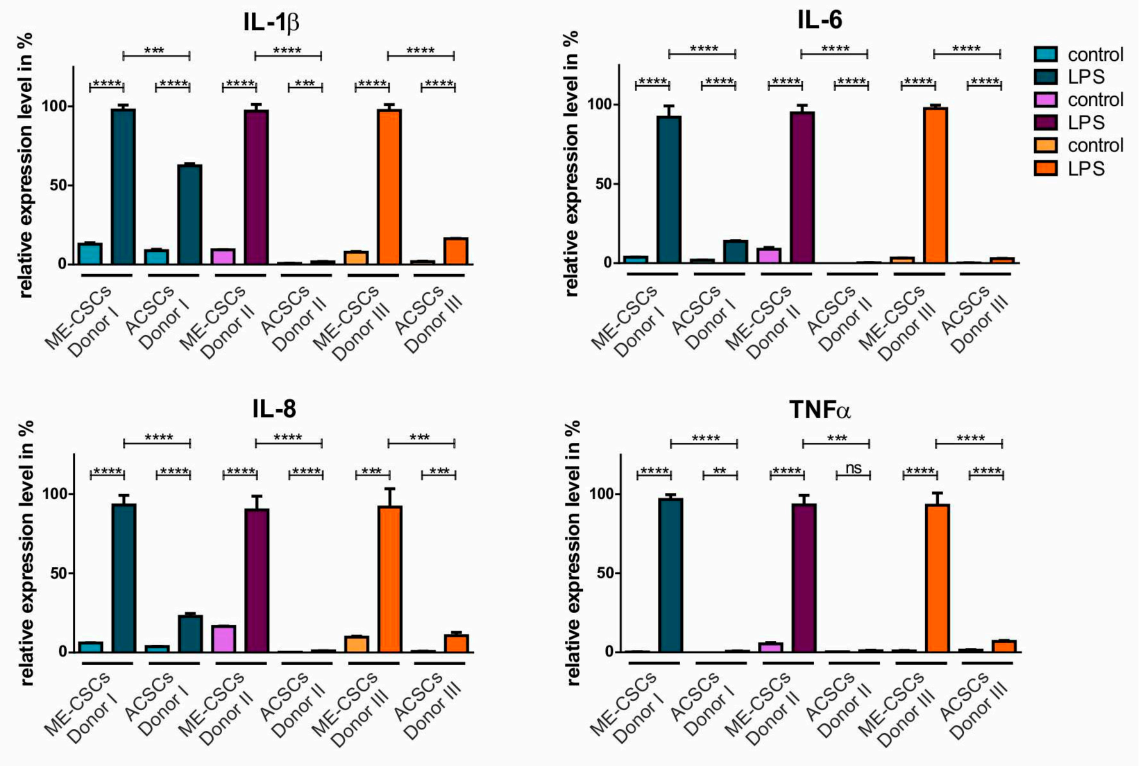

Figure S2. Expression levels of pro-inflammatory genes in ME-CSCs and ACSCs upon LPS-stimulation on single-donor level. Pro-inflammatory mediators IL-1ß, IL- 6, IL-8 and TNFα were strongly and significantly increased in all ME-CSCs populations derived from three donors after treatment with LPS from *S. enterica* compared to untreated control and LPS-treated ACSCs (****≤0.0001, ***≤0.001, ns>0.05, unpaired t-test, one-tailed, confidence interval: 95%).

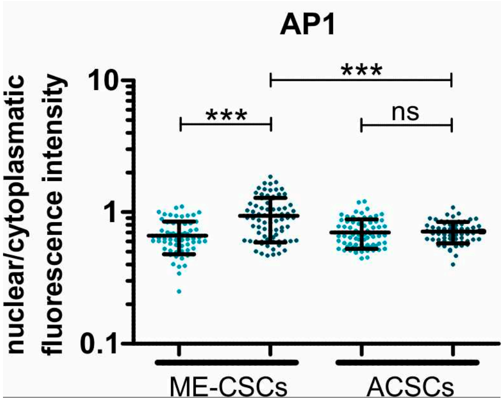

Figure S3. Significant translocation of AP1 protein in ME-CSCs upon LPS-treatment. Quantification of nuclear/cytoplasmatic fluorescence intensity of immunocytochemical stainings revealed significantly increased nuclear protein amounts of AP-1 in LPS-treated ME-CSCs compared to control and ACSCs exposed to LPS (LPS from *S. enterica*, ***≤0.001, ns>0.05, Mann Whitney test, one-tailed, confidence interval: 95%).

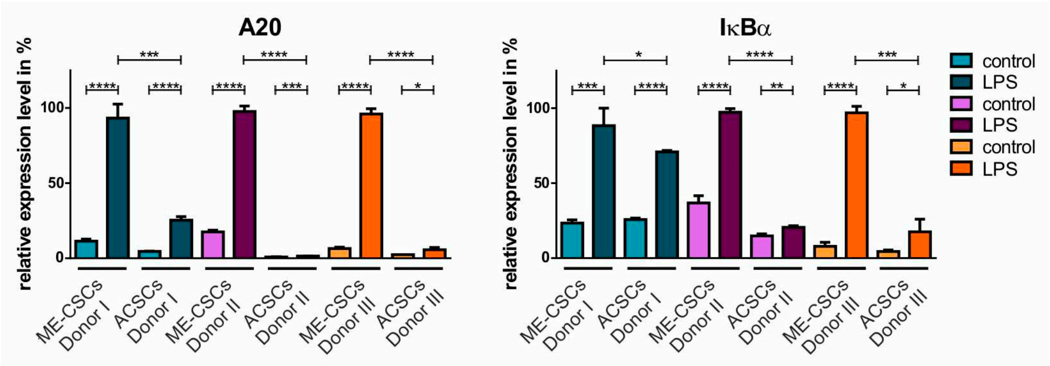

Figure S4. Expression levels of NF-κB target genes A20 and IκBα in ME-CSCs and ACSCs upon LPS-stimulation on single-donor level. NF-κB target genes A20 and IκBα were strongly and significantly increased in all ME-CSCs populations derived from three donors after treatment with LPS from compared to untreated control and LPS-treated ACSCs (****≤0.0001, ***≤0.001, **≤0.01, *≤0.05, unpaired t-test, one-tailed, confidence interval: 95%).

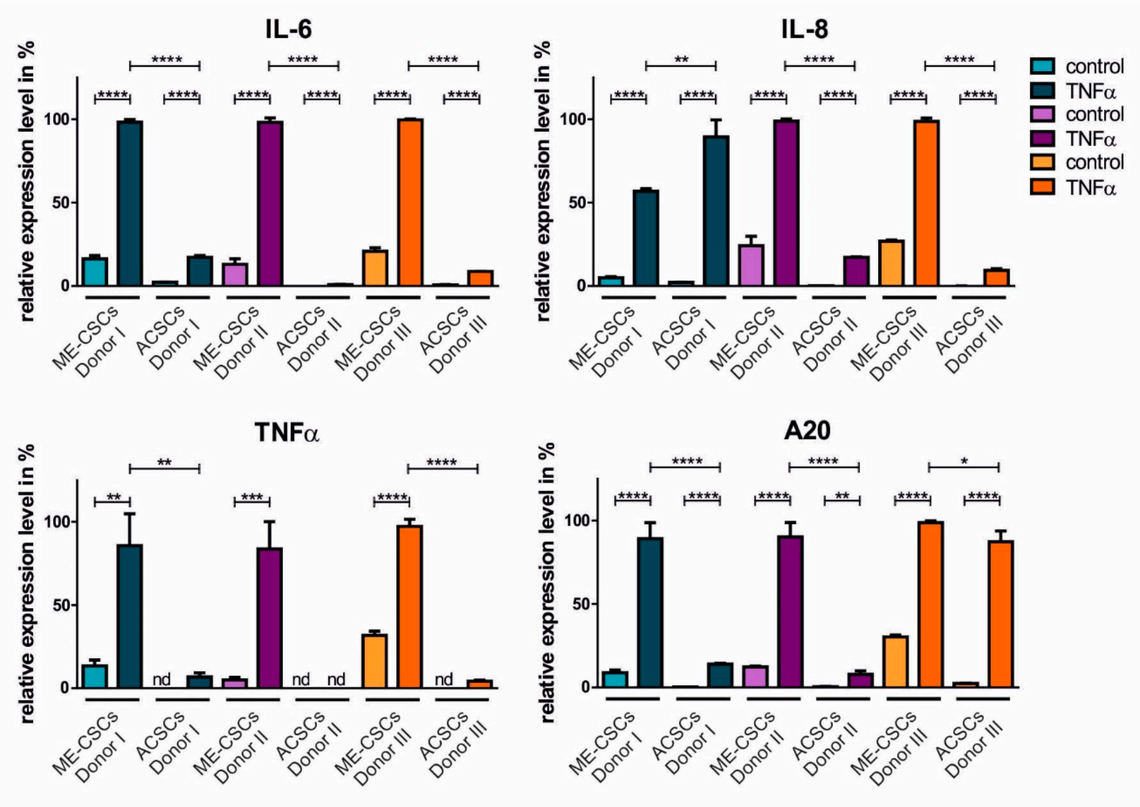

Figure S5. ME-CSCs show a TNF*α*-mediated feed-forward-loop of pro-inflammatory NF-κB target gene expression on single-donor level. qPCR analysis revealed a significant increase in the expression levels of IL-6, IL-8, TNF*α* and A20 in ME-CSCs stimulated with TNF*α* compared to control and TNF*α*-stimulated ACSCs (****≤0.0001, ***≤0.001, **≤0.01, *≤0.05, unpaired t-test, one-tailed, confidence interval: 95%, nd: not detectable).
